# The biological basis for current treatment strategies for granulomatous disease in common variable immunodeficiency

**DOI:** 10.1097/ACI.0000000000001032

**Published:** 2024-10-21

**Authors:** Astrid C. van Stigt, Giulia Gualtiero, Francesco Cinetto, Virgil A.S.H. Dalm, Hanna IJspeert, Francesco Muscianisi

**Affiliations:** a Laboratory Medical Immunology, Department of Immunology; b Division of Allergy & Clinical Immunology, Department of Internal Medicine, Erasmus University Medical Center Rotterdam, Rotterdam, The Netherlands; c Hematology and Clinical Immunology Unit, Department of Medicine (DIMED); d Veneto Institute of Molecular Medicine (VIMM); e Rare Diseases Referral Center, Internal Medicine 1, Department of Medicine (DIMED), AULSS2 Marca Trevigiana, Ca’ Foncello Hospital, University of Padova, Padova, Italy

**Keywords:** common variable immunodeficiency, granuloma, granulomatous lymphocytic interstitial lung disease, immunomodulatory, treatment

## Abstract

**Purpose of review:**

The pathogenesis of granulomatous disease in common variable immunodeficiency (CVID) is still largely unknown, which hampers effective treatment. This review describes the current knowledge on the pathogenesis of granuloma formation in CVID and the biological basis of the current treatment options.

**Recent findings:**

Histological analysis shows that T and B cells are abundantly present in the granulomas that are less well organized and are frequently associated with lymphoid hyperplasia. Increased presence of activation markers such as soluble IL-2 receptor (sIL-2R) and IFN-ɣ, suggest increased Th1-cell activity. Moreover, B-cell abnormalities are prominent in CVID, with elevated IgM, BAFF, and CD21low B cells correlating with granulomatous disease progression. Innate immune alterations, as M2 macrophages and neutrophil dysregulation, indicate chronic inflammation. Therapeutic regimens include glucocorticoids, DMARDs, and biologicals like rituximab.

**Summary:**

Our review links the biological context of CVID with granulomatous disease or GLILD to currently prescribed therapies and potential targeted treatments.

## INTRODUCTION

**FIGURE 1:**
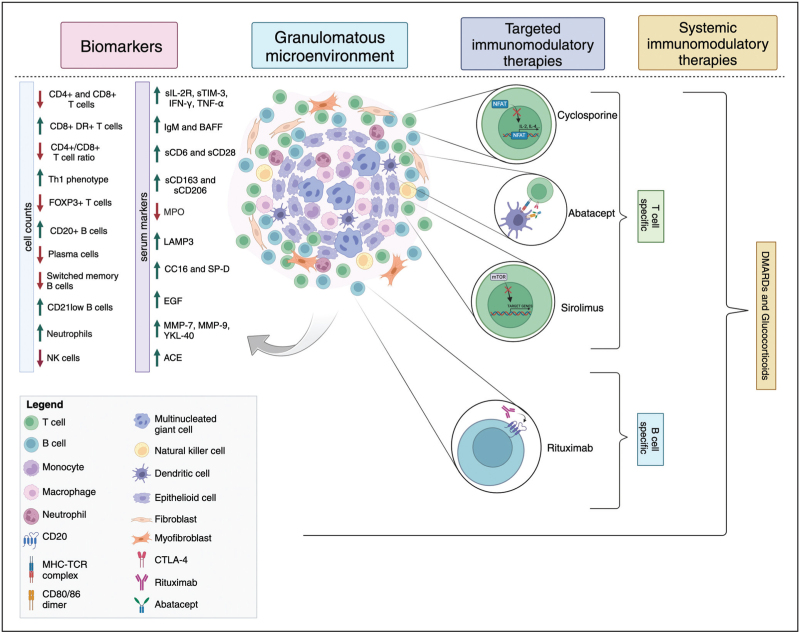
Comprehensive representation of described topics of this review. The left side shows alterations in blood or bronchoalveolar-lavage fluid biomarkers, followed by histologic organization of the granulomatous microenvironment. Targeted and general immunomodulatory therapies for granulomatous lymphocytic interstitial lung disease (GLILD) or granulomatous disease in common variable immunodeficiency are depicted on the right side. The target therapies section highlights the mechanisms of action for T-cell-specific and B-cell-specific treatments represented by cyclosporine, abatacept, sirolimus, and rituximab. Furthermore, the general therapies section indicates the use of disease-modifying antirheumatic drugs (DMARDs; azathioprine, methotrexate, mycophenolate mofetil, anti-TNF-α agents) and glucocorticoids as general immunomodulating therapies affecting a broad cellular spectrum. Image created with BioRender.com.

Granulomatous disease in common variable immune deficiency (CVID), including granulomatous lymphocytic interstitial lung disease (GLILD), is a rare but severe noninfectious complication, occurring in up to 8–22% of patients with CVID [1–3]. At present, standardized treatment regimens are lacking [1–8,9^▪▪^]. Granulomas typically form in response to hard-to-clear antigenic triggers. However, for granulomatous disease in CVID, this trigger remains unknown. Uncontrolled inflammation and granuloma formation may require immunomodulatory therapies, including glucocorticoids, disease-modifying antirheumatic drugs (DMARDs) and biologicals like rituximab, with varying effects [3,7,8,9^▪▪^,^10^]. To provide a basis for targeted therapies, it is important to correlate the pathophysiology of the disease to current and potential future treatments. With this review, we aim to give an overview of and correlation between the pathophysiology of granulomatous disease in CVID and current and future immunomodulatory treatments (Fig. 1). 

**Box 1:**
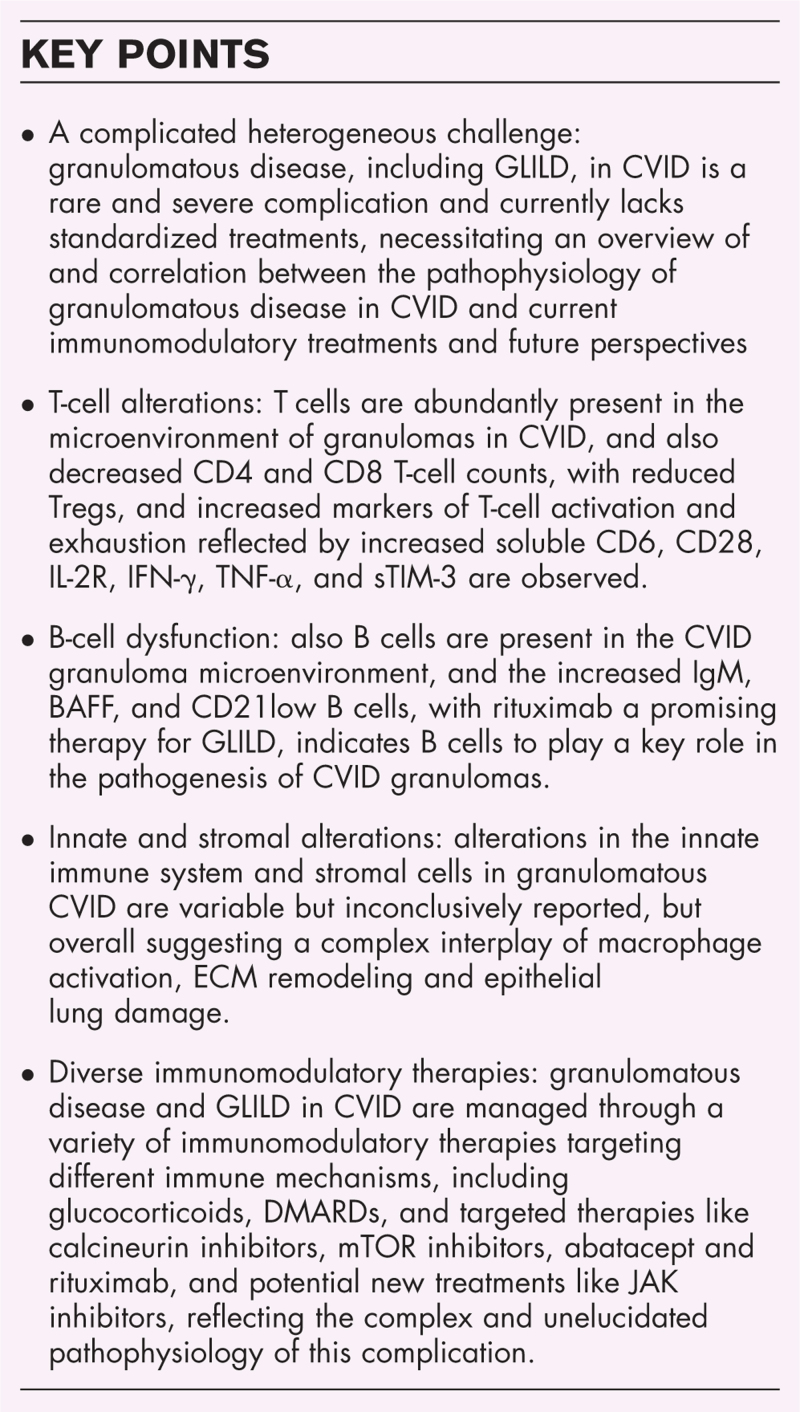
no caption available

## HISTOLOGICAL ORGANIZATION OF GRANULOMAS IN COMMON VARIABLE IMMUNODEFICIENCY

Granulomas are typically spherical in shape, with a core consisting of tissue-resident macrophages, which eventually can form multinucleated giant cells by fusion, and T cells encircling the granuloma [9^▪▪^,^11^–^13^,^14^^▪▪^,^15^,^16^]. However, for granulomas and GLILD in CVID, limited and varying histological observations are reported [9^▪▪^,^12^,^13^,^16^–^19^]. In CVID, granulomas seem to be poorly circumscribed and might appear with several conformations [12,13,17]. CVID granulomas are nonnecrotizing, and consist of epithelioid histiocytes with, additionally, a reduced subset of multinucleated giant cells [12]. Most GLILD histology specimens exhibit organizing pneumonia, which is characterized by intra-alveolar buds of granulation tissue, including myofibroblasts and connective tissue [12,16].

Apart from the reduced organization, granulomas in GLILD also appear randomly dispersed throughout the lung parenchyma, and remarkably occur both distant from inflammatory infiltrates, as well as in conjunction with lymphocytic infiltrations [12]. Lymphoid proliferation is a key feature of GLILD, presenting with differential patterns of pulmonary lymphoid hyperplasia, such as follicular bronchiolitis, lymphocytic interstitial pneumonitis, and nodular lymphoid hyperplasia [16,17,20,21]. Sheets of lymphocytes have been observed in most cases of diffuse lymphocytic infiltrations [13]. The lymphoid hyperplasia, being epithelioid granulomas organized in a crown shape around a reactive follicle or encircled by a dense lymphocytic infiltrate, seems to sustain the granulomatous inflammation [22^▪^]. Diffuse interstitial inflammation has also been linked to poorly formed granulomas [12]. Immunohistochemistry in CVID+GLILD samples revealed heterogeneity in lymphocytic infiltrates. In a small number, CD20+ B cells surrounded by T cells were detected. On the other hand, T cells can spread out and localize in areas where there are no B cells. Regarding T-cell composition in histological samples, the percentage of CD4+ T cells was consistently present when they additionally encircled B-cell follicles. In other instances, distinct pathogenic entities were highlighted by an equal percentage of CD4+ and CD8+. Furthermore, several histological sections containing B-cell follicles showed to exhibit proliferating germinal centers [12,13,17]. Pulmonary B-cell hyperplasia is a significant characteristic of CVID+GLILD [17]. These findings reveal the complex interplay between B cells and T cells in CVID granulomas and underscore the key role of B-cell dysregulation in the disease.

## THE INNATE IMMUNE AND STROMAL CELL CONTRIBUTION TO COMMON VARIABLE IMMUNODEFICIENCY GRANULOMAS

Peripheral blood white blood cell (WBC), monocyte, and neutrophil counts typically do not show differences between CVID subgroups [23]. However, progressive granulomatous CVID patients showed a significant decrease in WBC compared with CVID with only infections [24^▪^]. The increased serum sCD163 and increased sCD206 levels in CVID+GLILD and progressive granulomatous disease suggest M2-phenotype macrophages’ involvement, fitting a chronic inflammation state [24^▪^,^25^].

For CVID in general, decreased expression of the neutrophil surface markers CD11b, CD15 and CD16 with defective neutrophil function, and elevated levels of cytokines associated with granulocyte–macrophage lineage activation are observed [26,27]. Also, increased serum elastase and myeloperoxidase (MPO) levels are detected in CVID patients with splenomegaly, a clinical feature more frequently observed in CVID patients with GLILD [28–30]. Specifically in progressive CVID+GLILD, slightly increased neutrophil counts versus stable or no GLILD are reported [23]. However, slightly decreased MPO levels in serum of CVID+GLILD patients versus CVID with other complications or only infections are also reported [25].

Natural killer (NK) cell counts are clearly diminished in CVID patients with granulomatous disease compared with those without, although reduced NK cell counts may not be specific to granulomatous disease as it is also associated with other noninfectious complications [6,31]. In BALF of sarcoidosis patients, an increased neutrophil and NK count seems associated with a worse clinical outcome, bearing in mind, no significant differences in neutrophil counts between sarcoidosis and CVID+GLILD are observed [14^▪▪^,^32^,^33^]. Elevated serum levels of pulmonary epithelial markers LAMP3, CC16, and SP-D are noted in GLILD [25,34–37]. The Increased EGF, MMP-7, MMP-9, and YKL-40 levels in serum of CVID+GLILD indicate extra cellular matrix remodeling [25,27,38]. Angiotensin-Converting-Enzyme (ACE), mainly produced by endothelial cells, but possibly also by alveolar macrophages and epithelial cells, is elevated in CVID granulomatous disease [24^▪^,^32^,^39^,^40^]. Overall, this highlights the involvement of nonimmune cellular compartments in granuloma formation in CVID.

## THE ADAPTIVE IMMUNE CELL CONTRIBUTION TO COMMON VARIABLE IMMUNODEFICIENCY GRANULOMAS

CVID is characterized by a defect in the differentiation or dysfunction of B cells [4]. Interestingly, CVID patients with granulomatous disease have higher levels of IgM, B-cell-activating factor (BAFF), and CD21low B cells compared with CVID patients with infections only. The elevated serum IgM levels correlate with granulomatous disease progression and pulmonary B-cell hyperplasia [23,41]. Possibly, a limited number of plasma cells with a predominant IgM isotype present at the granulomatous inflammatory sites are the source [42].

BAFF is produced by innate leukocytes, fibroblasts, or possibly activated T cells, is upregulated by IFN-ɣ:STAT1 signaling [18], and binds to the BAFF receptor on naive B cells. BAFF-mediated apoptosis resistance can lead to increased immature B cells, and might contribute to granulomatous disease in CVID [43]. However, conflicting reports exist on BAFF levels and other correlated markers in sera or bronchoalveolar-lavage fluid (BALF). Maglione *et al.*[23] detected increased levels of BAFF, in serum and lung biopsies of progressive CVID+GLILD patients, which was associated with B-cell hyperplasia and germinal center formation in lung tissues, and correlated with increased serum IgM levels. The levels of A proliferation-inducing ligand (APRIL) were not different, but they detected reduced levels of TACI and B-cell maturation antigen (BCMA) in line with the impaired maturation of B cells [23]. Oppositely, others reported nonsignificant increased BAFF in sera or BALF, with an increase of APRIL in the BALF [25,33]. Also, a significant increase of soluble BCMA in CVID+GLILD compared with CVID with other noninfectious complications was reported [25]. This is intriguing as sBCMA is shed from plasma cells, and plasma cells are generally reduced in CVID patients [18,44]. However, unlike serum IgM levels, the sBCMA levels were not different between progressive CVID+GLILD versus resting GLILD [23,25]. Class-switched memory B cells are significantly lower in CVID patients with granulomatous disease compared with other CVID patients [1,5,6,25,28,33], whereas CD21low B cells are increased in CVID+GLILD, and correlate with lung germinal centers [5,9^▪▪^,^19^,^25^,^28^,^33^,^42^,^45^,^46^]. As CD21low B cells have low CD83 expression, with retained CD19 and IgM expression, they are considered as preactivated, polyclonal B cells that are potentially autoreactive and functionally attenuated [45–47]. Their precise role in the pathogenesis of granulomatous CVID or GLILD remains under investigation. Of note, although Fraz *et al.*[25] report a significant increase of CD21low B cells without a significant difference in follicular helper T (Tfh cells) in their CVID+GLILD cohort. Importantly, CD20 is expressed on CD21low B cells, as on pre-B and other mature B cells excluding plasma cells, and is a promising druggable target [46,48].

CVID patients with GLILD or progressive granulomatous disease have decreased CD4+ and CD8+ T-cell counts in peripheral blood compared with those without granulomatous disease, suggesting T-cell migration to the granulomatous lesions [5,6,9^▪▪^,^12^,^13^,24^▪^,^25^,^41^,^42^,^49^]. Within the CD8+ T-cell reduction, a larger proportion of memory and senescent cytotoxic T cells is reported [6]. However, an increase in CD8+DR+ T cells with an active cytotoxic phenotype and reduced TCR repertoire diversity have been observed in the blood of CVID patients with noninfectious complications, including granulomatous disease [50]. In CVID+GLILD patients, increased serum levels of CD6, CD28, soluble IL-2 receptor (sIL-2R), IFN-γ, and TNF-α suggest activation of CD4+ T cells with Th1 phenotype [23,25,34,51]. Also, the combined increase of sTIM-3 and sIL2R is indicative for T-cell exhaustion [34]. Elevated sIL-2R and IFN-ɣ levels in CVID+GLILD, especially with progressive disease, suggests CD4+ T-cell activation [23,24^▪^,^39^,^51^]. Of note, dendritic cells, monocytes and B cells can also release sIL-2R [52]. Regulatory T cells (Tregs) are reported to be reduced in CVID with granulomatous disease [1,3,9^▪▪^,^12^,^25^,^33^,^53^], and despite increased IL-10 and CD83 levels, a lack of increased TGF-β suggests a less effective Treg function in GLILD [34]. Tfh cells, specialized in B-cell help, are increased in CVID patients with noninfectious complications [51,54]. Le Saos-Patrinos *et al.*[54] hypothesize that switched memory B cells in CVID patients, despite their low levels, contribute to autoimmunity, with Tfh aiding in autoimmune manifestations through their role as switched memory B-cell inducers. However, conflicting results exist regarding Tfh-cell levels in CVID patients with granulomatous disease or GLILD [25,51].

## MANAGEMENT OF GRANULOMATOUS COMPLICATIONS IN COMMON VARIABLE IMMUNODEFICIENCY-AFFECTED PATIENTS

As discussed above, granulomatous inflammation and lymphocytic infiltration are associated with altered innate and adaptive immunity [55]. Here, we discuss the biological basis of the currently used immunosuppressive drugs to treat granulomatous disease and GLILD in CVID.

### Glucocorticoids

Glucocorticoids exert profound immunomodulatory effects by both nongenomic and genomic effects, which possibly target every cell type involved in granulomatous inflammation [56]. Rapid nongenomic effects do not require protein synthesis [57]; the binding of glucocorticoids to glucocorticoid receptor also liberates accessory proteins that participate in secondary signaling cascades, which lead, for example, to the inhibition of phospholipase A2 activity and a decreased release of arachidonic acid, the main precursor of inflammatory mediators [58,59]. Furthermore, short-term exposure to glucocorticoids can promote apoptosis of lymphocytes [60,61], macrophages [62], and dendritic cells [63].

Nonrapid genomic effects are mediated by glucocorticoids-glucocorticoid receptor complex and its binding to glucocorticoid-responsive elements (GREs) of the DNA, enhancing/reducing targeted gene expression [56]. Glucocorticoids inhibit the expression of main pro-inflammatory cytokines and enhance the expression of anti-inflammatory ones, like IL-10 [64]. Furthermore, glucocorticoids downregulate various transcription factors like NF-kB and NFAT, which are crucial in antigen-driven TCR signaling [65]. The 2017 consensus statement on the definition, diagnosis, and management of GLILD addresses glucocorticoid monotherapy as the first-line treatment [4] despite limited evidence and common relapses after discontinuation [7,8]. However, Smits *et al.* presented a more convincing description of the efficacy and safety of first-line high-dose glucocorticoid monotherapy versus watchful waiting, showing significant improvement in lung imaging and function, with 72% maintaining remission for at least 2 years. Low-dose maintenance therapy did not improve remission rates, and retreatment after relapse was generally ineffective, suggesting the need for other immunosuppressive regimens after relapse [66^▪^].

### Traditional disease-modifying anti-rheumatic drugs

Azathioprine (AZA) is a pro-drug whose active metabolites act like nucleoside analogues and inhibit purine synthesis, thus halting division and inhibiting protein synthesis of highly proliferative cells, like leukocytes [67]. AZA has been reported as effective in combination with glucocorticoids, GC+RTX, RTX in CVID with GLILD [8], as well as in combination with glucocorticoids in a single case of selective skin granulomatous involvement [68].

Methotrexate (MTX) inhibits dihydrofolate reductase and reduce the synthesis of tetrahydrofolate, a central element for the de novo synthesis of purine nucleotides and some amino acids (serine and methionine) in highly replicating cells. MTX low doses also interfere with adenosine metabolism, which leads to a reduced synthesis of pro-inflammatory cytokines and a reduced activation of T cells and monocytes, via downregulated NFkB signaling [69]. Few studies reported MTX use, mainly with other immunosuppressive drugs (glucocorticoids) and generally associated with remission [7].

Mycophenolate mofetil (MMF) is the pro-drug of mycophenolic acid, which inhibits inosine monophosphate dehydrogenase, blocking purine nucleotide synthesis. Although other cell types can use salvage pathways, B cells and T cells strictly depend on this biosynthetic source, thus explaining MMF's immunosuppressive activity. MMF also inhibits the glycosylation and expression of adhesion molecules, impairing leukocyte diapedesis [70]. Few studies reported the use of MMF in CVID with granulomatous disease, overall associated with remission, either as monotherapy or as a maintenance therapy after glucocorticoid or RTX induction therapy [7,71,72].

Anti-TNF-α agents are mAbs (e.g. infliximab and adalimumab) or fusion proteins (etanercept) which bind and inhibit soluble TNF-α, an important mediator of Th cells and macrophages interaction in the granuloma formation [72]. These drugs represent a cornerstone in refractory sarcoidosis [73]. Infliximab monotherapy is described as effective mainly in case of extrapulmonary involvement, inducing disease remission [74–79]. Etanercept monotherapy was instead described only in three patients with granulomatous skin involvement, all achieving remission [80–82].

### T-cell-targeting therapies

Nuclear factor of activated T cells (NFAT) is one of the crucial factors for T-cell responses. Calcineurin, a phosphatase that activates NFAT, allows its nuclear translocation, where it promotes the transcription of IL-2 – the most important mitogen and activator of T cells – IL-4, INF-γ, and TNF-α. Cyclosporine binds to the cytosolic protein cyclophilin, tacrolimus to a cytosolic protein called FKBP; both these complexes inhibit calcineurin, thus exerting a potent T-cell-selective cytostatic and immunosuppressive effect [83,84]. There are few reports on the efficacy of cyclosporine in inducing GLILD remission, after failure of glucocorticoid treatment [85,86]. No reports concerning tacrolimus are available. A patient with severe granulomatous liver disease, initially treated with cyclosporine and MMF, showed recurrence in the liver posttransplant and was switched to sirolimus [6].

The mechanistic target of rapamycin (mTOR)-signaling pathway senses and integrates environmental signals to regulate metabolism and growth in many cell types via the regulation of glycolysis or mitochondrial metabolism, to influence effector responses. Activation of mTOR also regulates inflammatory responses in innate immune cells, such as monocytes, macrophages, and dendritic cells [87]. T cells rely on mTOR signaling [88] and TCR activation causes mTOR activation and allows it to regulate the transition from the G1 to the S phase of the cell cycle [89]. In addition, once T cells are activated by IL-12, mTOR may drive the development of Th1 cells by stimulating the production of IFN-γ [90]. Few case reports showed the efficacy of mTOR inhibitor sirolimus in the treatment of GLILD, suggesting a possible role of mTOR pathway in GLILD as shown for sarcoidosis [87]. Deyà-Martínez *et al.*[91] reported the case of a boy who relapsed after RTX treatment, achieving remission once switched to sirolimus monotherapy.

Cytotoxic T-lymphocyte antigen-4 (CTLA-4) is an inhibitory receptor constitutively expressed on Treg and induced in activated T cells [92]. CTLA-4 binds co-stimulatory receptors CD80/CD86 and removes them from the surfaces of APCs through trans endocytosis, resulting in reduction in APC-mediated activation of conventional T cells, preventing prolonged immune response and promoting immune tolerance [93]. Lipopolysaccharide-responsive beige-like anchor (LRBA) binds to the cytoplasmic tails of CTLA-4 and prevents the trafficking of CTLA-4 to lysosomes and consequent degradation [94]. Abatacept consists of the Fc region of immunoglobulin IgG1 fused to CTLA-4 [95] and thus prevents excessive T-cell proliferation in CVID patients with CTLA-4 haploinsufficiency/LRBA deficiency. A total of three case series described the use of abatacept for the treatment of CTLA-4/LRBA-mutated GLILD patients, and all reported an improvement of clinical symptoms and radiological clinical findings [90,92,96^▪▪^]. A recent prospective, open-label, nonrandomized phase II trial reported the efficacy of abatacept even in a cohort of 10 GLILD patients with genetically undefined CVID after first-line therapy with glucocorticoids [92], basing on the previous observation that GLILD patients often exhibit increased circulating CD21low B cells expressing high levels of CD86 and decreased FOXP3+ regulatory T cells [92,93]. Moreover, further observations emphasize the role of CTLA-4 in immune dysregulation of other granulomatous diseases, like sarcoidosis, whose patients present decreased CTLA-4 expression on regulatory T cells [81]. Of note, blocking CTLA-4 to breach immune tolerance in cancer therapy can lead to granulomatous disease-mimicking sarcoidosis [82–84].

### B-cell-targeting therapies

RTX is a chimeric mAb targeting the transmembrane protein CD20 of pre-B, mature B cells, and plasma blasts, thus resulting in a profound peripheral B-cell depletion. CD20 functions are not completely understood, but it seems to regulate an early step in the activation process for cell cycle initiation and differentiation [97]. RTX induces B-cell killing by: NK cells through antibody-dependent cellular cytotoxicity (ADCC); membrane attack complex via complement activation (complement-mediated cell cytotoxicity); and reticulo-endothelial system cells via opsonization and consequent phagocytosis [98].

In line with the emerging evidences on the role of B-cell hyperplasia in driving and maintaining GLILD [23], several authors reported the efficacy of RTX, both as a single agent [99–103] and in combination with other immunosuppressive drugs: glucocorticoids [104], AZA [16,105], and MMF [105,106]. Tessarin *et al.*[19] reported the efficacy and safety of RTX monotherapy in a cohort of six GLILD patients, assessing a reduction of symptom burden and an improved quality of life compared with a control group of CVID patients without GLILD, as well as a significant improvement of TLC and DLCO and a restoration of CT scan findings.

### Potential therapies

A wide range of cytokines (INF-γ, IL-2, IL-6, IL-12, IL-18) – mainly produced by CD4+ T cells and macrophages – allow immune cells’ communication, thus being central elements in sustaining granulomatous inflammation. Many of these cytokines’ biological actions rely on the JAK-STAT pathway [107]. Analysis of tissue and circulating mononuclear cells from patients with sarcoidosis consistently revealed a constitutive JAK-STAT activation and immunohistochemistry showed constitutive activation of STAT1 in granuloma macrophages and STAT3 in surrounding lymphocytes [108,109]. Different authors reported a dramatic improvement in cutaneous and/or internal organ sarcoidosis patients with JAK inhibitors, including ameliorated lung function in case of pulmonary involvement [109,110]. Therefore, the use of JAK inhibitors could be hypothesized even in granulomatous complications of CVID, despite no reports are currently available.

## CONCLUSION

Predicting individual patient responses to immunomodulatory therapies remains challenging. Genetic screening identified useful genetic alterations [4,5,9^▪▪^]. However, mostly genetic variants are not found or the functional meaning is uncertain [2,9^▪▪^]. Methods to functionally assess cellular or protein alterations in individual patients could advance personalized precision medicine. An in-vitro model, using patient-derived cells, examining granuloma formation and drug testing, could be promising, something that is already further advanced in the sarcoidosis field [111]. Overall, more in-depth knowledge regarding the pathophysiology and immunomodulatory treatment response of granulomas in CVID is of the utmost importance to clinically advance further.

## Acknowledgements

*None.*

### Financial support and sponsorship

*This study was funded by the Jeffrey Modell Foundation (JMF) awarded to H.I.J. and Erasmus MC2 Young Investigator Grant awarded to H.I.J. G.G., F.C., and F.M. were supported by the Foundation ‘FoRiBiCa ETS’ (Fondazione per la Ricerca Biomedica Cardiovascolare e le Malattie Rare Ente del Terzo Settore).*

### Conflicts of interest

*There are no conflicts of interest.*

## REFERENCES AND RECOMMENDED READING

Papers of particular interest, published within the annual period of review, have been highlighted as:

▪ of special interest
▪▪ of outstanding interest
